# Bioinformatic Analysis Identifies of Potential miRNA-mRNA Regulatory Networks Involved in the Pathogenesis of Lung Cancer

**DOI:** 10.1155/2022/6295934

**Published:** 2022-09-30

**Authors:** Dexun Hao, Yanshuang Li, Jiang Shi, Junguang Jiang

**Affiliations:** ^1^Department of Geriatric Respiratory and Sleep, The First Affiliated Hospital of Zhengzhou University, Zhengzhou 450000, Henan, China; ^2^Department of Anesthesiology, The First Affiliated Hospital of Zhengzhou University, Zhengzhou 450000, Henan, China

## Abstract

**Objective:**

The purpose of the present study was to explore the biomarkers related to lung cancer based on the bioinformatics method, which might be new targets for lung cancer treatment.

**Methods:**

GSE17681 and GSE18842 were obtained from the Gene Expression Omnibus (GEO) database. The differentially expressed miRNAs (DEMs) and genes (DEGs) in lung cancer samples were screened via the GEO2R online tool. DEMs were submitted to the mirDIP website to predict target genes. Gene Ontology (GO) analysis and Kyoto Encyclopedia of Genes and Genomes (KEGG) analysis were conducted via uploading DEGs to the DAVID database. The protein-protein interaction network (PPI) of the DEGs was analyzed by STRING's online tool. Then, the PPI network was visualized using Cytoscape 3.8.0.

**Results:**

46 DEMs were identified in GSE17681, and the website predicted that there were 873 target genes of these DEMs. 1029 DEGs were identified in the GSE18842 chip. GO analysis suggested that the co-DEGs participated in the canonical Wnt signaling pathway, regulation of the Wnt signaling pathway, a serine/threonine kinase signaling pathway, the Wnt signaling pathway, and cell-cell signaling by Wnt. KEGG analysis results showed the co-DEGs of GSE17681 and GSE18842 were related to the Hippo signaling pathway and adhesion molecules. In addition, six hub genes that were related to lung cancer were identified as hub genes, including mTOR, NF1, CHD7, ETS1, IL-6, and COL1A1.

**Conclusions:**

The present study identified six hub genes that were related to lung cancer, including mTOR, NF1, CHD7, ETS1, IL-6, and COL1A1, which might be a potential target for lung cancer.

## 1. Introduction

At present, the incidence rate and mortality of lung cancer rank first in China [1], while non-small cell lung cancer (NSCLC) is the most common type of lung cancer, accounting for about 85% of them [2]. Adenocarcinoma and squamous cell carcinoma are common pathological types of NSCLC [3]. Because there are no obvious clinical symptoms in the early stage, the vast majority of NSCLC patients have entered the late stage at the time of diagnosis, and the 5-year survival rate is about 15%. Radiotherapy, chemotherapy, and surgery play a key role in the treatment of NSCLC, but 50% of lung cancer patients die of tumor recurrence [4]. Therefore, it is very important to find biomarkers that can accurately predict the prognosis of patients. Elucidating the genetic changes related to the occurrence and development of the disease at the molecular level will be conducive to the diagnosis, treatment, and prognosis of the disease. At present, the pathogenesis of NSCLC is still not clear, so mining the genetic changes related to NSCLC from the genome level will likely provide more molecular markers for its diagnosis and prognosis.

MiRNA is a small noncoding RNA that can regulate gene expression by targeting mRNA. It is closely related to cell proliferation, differentiation, migration, and invasion and is involved in tumor development. The development of high-throughput technology provides abundant gene expression profile data for the study of the pathogenesis of NSCLC [5]. Many scholars have conducted in-depth studies on the pathogenesis of NSCLC at the gene level and found that miRNA is related to the occurrence and progression of NSCLC [6, 7]. In recent years, more and more researchers have begun to study NSCLC-targeted new drug development. However, most patients will develop resistance to targeted drugs, resulting in poor efficacy for targeted drugs [8]. Therefore, finding new therapeutic targets for NSCLC is of great significance for improving the survival rate of NSCLC patients. Although there are reports on the screening of the DEGs in NSCLC, the results are also different due to the inconsistent number of samples in each study and the influence of confounding factors such as patient source, tumor stage, and grade.

Therefore, this study screened two NSCLC chip datasets (GSE17681 and GSE18842) to find common DEGs, aiming to provide data support for the precise treatment and prognosis of NSCLC.

## 2. Material and Methods

The microarray data from Gene Expression Omnibus (GEO) databases, GSE17681 and GSE18842 were utilized in this study. The dataset GSE17681, which was the miRNA profiling and based on the GPL570 platform, collected 17 NSCLC specimens and 19 normal tissues. The dataset GSE18842 collected 46 NSCLC specimens and 45 normal tissues, which were based on the GPL570 platform.

### 2.1. Identification of DEGs

Firstly, the GEO2R online tool was utilized to screen the differentially expressed miRNAs (DEMs) and genes between lung cancer samples and healthy samples. The “GEOquery” and “limma” packages in R software were utilized to analyze data tables. adj. *P* < 0.05 and |logFC| > 2 were set as the criteria for significant differences. DEMs were submitted to the mirDIP website (https://ophid.utoronto.ca/mirDIP/index.jsp#r) to predict target genes. The intersection of the DEGs in GSE17681 and GSE18842 was conducted to screen out the common DEGs. The heatmap based on top 10 and bottom 10 DEGs were created by the “pheatmap” package. A volcano map was generated using the “ggplot2” package.

### 2.2. Functional and Pathway Enrichment Analyses

The biological information annotation database David (https://david.ncifcrf.gov/) was used for Gene Ontology (GO) analysis and Kyoto Encyclopedia of Genes and Genomes (KEGG) analysis, including cell components, molecular functions, biological processes, and signal pathways. The “pheatmap” package of R software was used to visualize the enrichment analysis results.

### 2.3. Protein-Protein Interaction Network (PPI) Network Construction

The PPI network of the DEGs was analyzed using STRING (https://cn.string-db.org/). Then, the visualization of the PPI network was performed by Cytoscape 3.8.0, and the genes with a high level of connectivity with surrounding genes were selected as hub genes.

## 3. Results

### 3.1. Identification of DEGs

**Figure 1:**
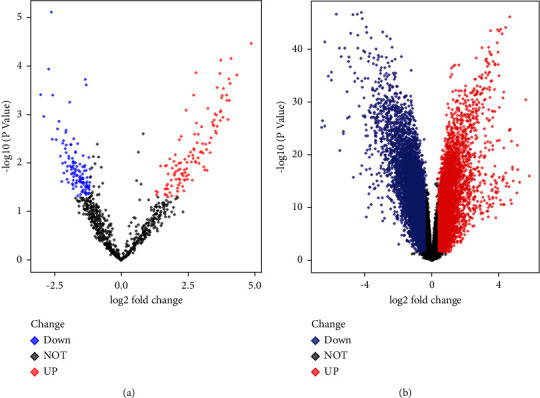
Volcano plots of the DEGs in GSE17681 (a) and GSE18842 (b).

**Figure 2:**
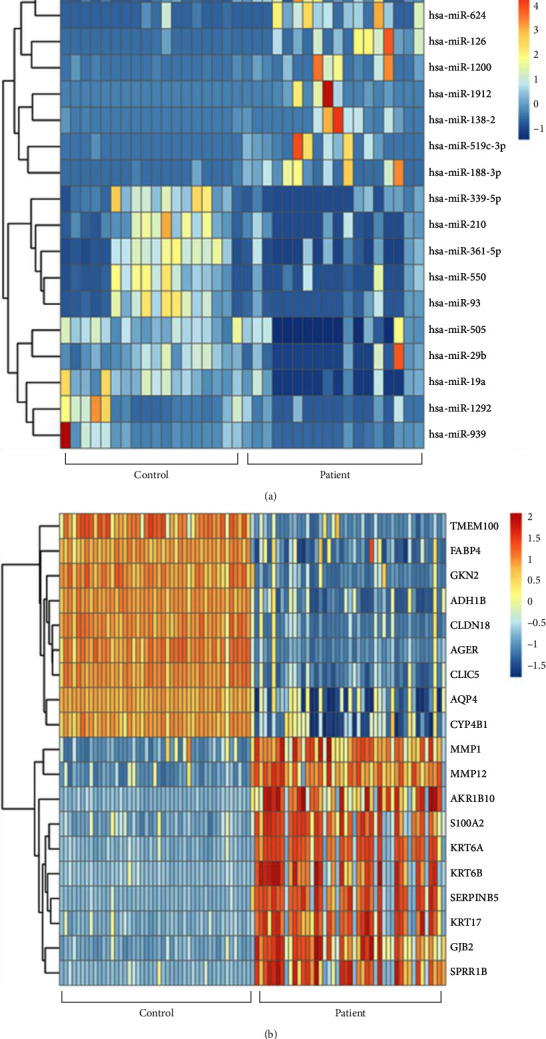
The heatmap plots of the top 10 genes. (a) GSE17681. (b) GSE18842.

The GSE17681 chip yielded 46 DEMs, of which 37 were upregulated and 9 were downregulated (Figure 1(a)). The website predicted that there were 873 target genes for these DEMs. 1029 DEGs were obtained on the GSE18842 chip, of which 419 were upregulated and 610 were downregulated (Figure 1(b)). Further analysis showed that 84 common genes (35 upregulated genes and 49 downregulated genes), which were named co-DEGs, were differentially expressed in lung cancer in these two-chip data. The top 10 and bottom 10 DEGs expressions were visualized by a heatmap (Figure 2).

### 3.2. GO Annotation Analyses of DEGs

**Figure 3:**
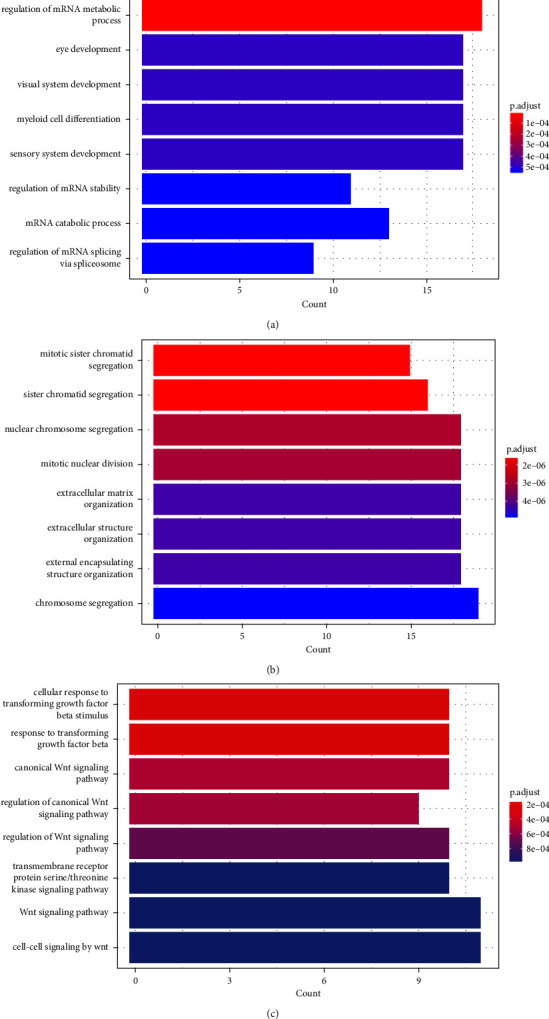
Gene Ontology (GO) analyses of the DEGs of GSE17681 (a), GSE18842 (b), and the co-DEGs (c).

The DEGs of GSE17681 participated in the regulation of mRNA metabolic process, eye development, visual system development, myeloid cell differentiation, sensory system development, regulation of mRNA stability, mRNA catabolic process, and regulation of mRNA splicing via spliceosome (Figure 3(a)). The DEGs of GSE18842 participated in mitotic sister chromatid segregation, sister chromatid segregation, and nuclear chromosome segregation (Figure 3(b)). The co-DEGs participated in the canonical Wnt signaling pathway, regulation of the canonical Wnt signaling pathway, regulation of the Wnt signaling pathway, the transmembrane receptor protein serine/threonine kinase signaling pathway, the Wnt signaling pathway, and cell-cell signaling by Wnt (Figure 3(c)).

### 3.3. KEGG Pathway Enrichment Analyses of DEGs

**Figure 4:**
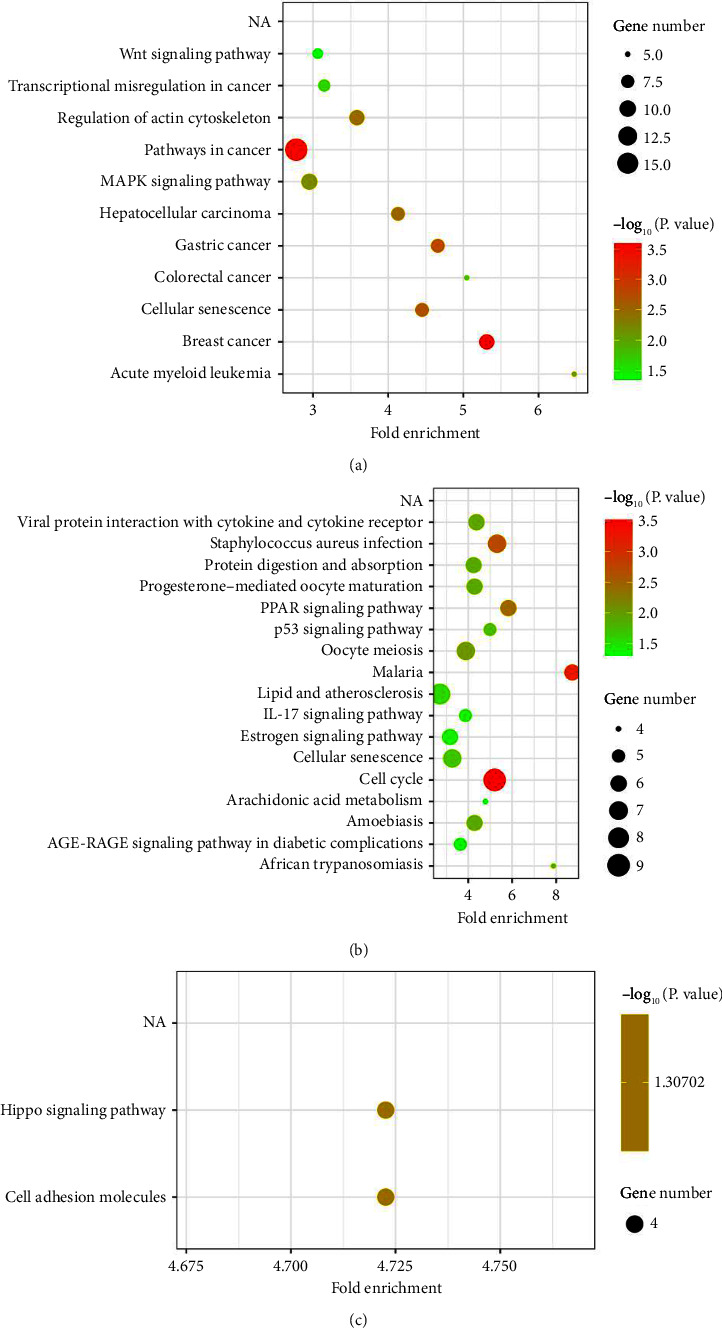
KEGG analyses of the DEGs of GSE17681 (a), GSE18842 (b), and the co-DEGs (c).

The DEGs of GSE17681 were related to the Wnt signaling pathway, hepatocellular carcinoma, acute myeloid leukemia, regulation of actin cytoskeleton, cellular senescence, pathways in cancer, the MAPK signaling pathway, gastric cancer, colorectal cancer, and breast cancer (Figure 4(a)). The DEGs of GSE18842 were related to protein digestion and absorption, progesterone-mediated oocyte maturation, PPAR signaling pathway, African trypanosomiasis, p53 signaling pathway, oocyte meiosis, malaria, lipid and atherosclerosis, IL-17 signaling pathway, estrogen signaling pathway, cellular senescence, cell cycle, arachidonic acid metabolism, amoebiasis, and the AGE-RAGE signaling pathway in diabetic complications (Figure 4(b)). The co-DEGs of GSE17681 and GSE18842 were related to the Hippo signaling pathway and adhesion molecules (Figure 4(c)).

### 3.4. PPI Network and Hub Genes

**Figure 5:**
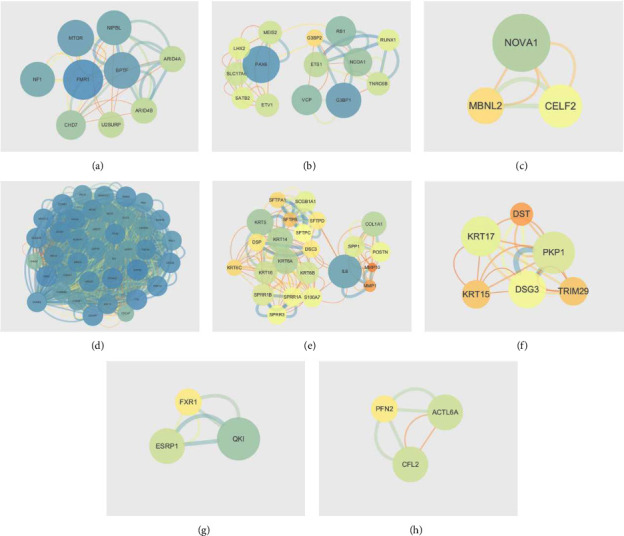
PPI networks are based on the screened DEGs. (a, b, c). The PPI networks are based on the DEGs of GSE17681 screened above. (d, e, f) The PPI networks are based on the DEGs of GSE18842 screened above. (g, h, i). The PPI networks are based on the co-DEGs of GSE17681 and GSE18842 screened above.

To identify the hub genes relevant to lung cancer, DEGs were uploaded to the STRING website to create a PPI network, and the visualization results are shown in Figure 5. Six hub genes were screened in relation to lung cancer, including mTOR, NF1, CHD7, ETS1, IL-6, and COL1A1.

## 4. Discussion

Over the years, although NSCLC treatment has been continuously improved, its 5-year survival rate has remained very low, at only 16% [9], mainly because its pathogenesis remains unclear. Lung cancer is a complex disease, and its occurrence may be a combination of polygenic and multipathway roles [10]. Therefore, finding key genes for lung carcinogenesis from the perspective of tumor genome-wide alterations may be an effective way to study the pathogenesis of lung cancer. With the rapid development of biological information technology, the application of high-throughput technologies such as microarray and whole genome sequencing to mine key genes in the process of disease occurrence and progression has brought new methods for exploring the molecular pathogenesis of the disease, improving clinical diagnosis, and targeting therapy [11]. At present, there are few studies on the pathogenesis and related molecular markers of NSCLC at home and abroad, which limits the timely diagnosis and treatment of NSCLC in clinical practice.

In this study, 46 DEMs (37 upregulated and 9 downregulated) were screened on the GSE17681 chip. The website predicted that there were 873 target genes for these DEMs. 1029 DEGs (419 upregulated and 610 downregulated) were screened on the GSE18842 chip. GO analysis results showed that the co-DEGs participated in the canonical Wnt signaling pathway, regulation of the canonical Wnt signaling pathway, the transmembrane receptor protein serine/threonine kinase signaling pathway, the Wnt signaling pathway, and cell-cell signaling by Wnt. KEGG analysis results showed the co-DEGs of GSE17681 and GSE18842 were related to the Hippo signaling pathway and adhesion molecules. Further verification found that six hub genes were screened in relation to lung cancer, including mTOR, NF1, CHD7, ETS1, IL-6, and COL1A1.

The activation of the mTOR signaling pathway is involved in the occurrence and development of human tumors and promotes the occurrence of tumors through a variety of mechanisms. The activation of various components in the mTOR signaling pathway is also a poor prognostic factor for many tumors. Inhibition of the mTOR signaling pathway can reverse drug resistance and improve the effect of chemotherapy and radiotherapy in vivo and in vitro [12]. MTOR was upregulated in numerous tumors, such as ovarian cancer, breast cancer, lung cancer, and so on [13]. The MTOR gene showed genome amplification in lung cancer and preinvasion bronchopathy, suggesting that the mTOR pathway was associated with the development of lung cancer [14]. The protein encoded by NF1 is associated with cell growth and differentiation. The NF1 gene is a tumor suppressor gene, and its encoded neurofibroma protein is a Ras GTP enzyme activator protein (RAS gap) [15]. Neurofibromatosis protein is functionally and structurally homologous to p120RasGAP. Gap proteins can terminate the Ras-mediated signal transduction pathway by activating the Ras-activated GTP binding form into an inactive GDP binding form. Therefore, the tumor suppressive function of NF1 is considered to be mainly dependent on its downregulation of the proto-oncogene RAS.

CHD7 is a member of the chromatin helicase DNA binding protein family. Its biological functions are mostly related to human congenital malformations [16], and it is abnormally expressed and activated in a variety of tumors such as SCLC and pancreatic cancer, regulating tumor cell proliferation, invasion, and other functions [17, 18]. Pleasance et al. found that CHD7 rearranged in NSCLC, which was relevant to abnormal cell damage repair, and played a certain role in promoting the progress of lung cancer [17]. ETS-1 is a proto-oncogene, which is positive in many tumors [19]. The ETS-1 gene is involved in cell growth and extracellular matrix invasion, which can promote tumor invasion and metastasis [20]. ETS-1 coding products constitute a large family of transcription regulators that participate in cell migration and apoptosis, angiogenesis, and organogenesis. ETS-1 is generally highly expressed in many malignant tumors. ETS-1 can also regulate some ECM target genes, including matrix proteins and other cell components involved in cell-matrix reactions [21]. When the expression of endogenous ETS-1 is inhibited, the expression of matrix-related proteins MMP-1 and MMP-2 is downregulated at the RNA level.

IL-6 is also a downstream effector of the oncogene RAS, which affects the immune regulation and hematopoietic regulation of the body [22] and promotes the differentiation and proliferation of T and B lymphocytes by activating target genes, so as to enhance the activity of neutrophils and monocytes and enhance the inflammatory response of local tissues. COL1A1 can form collagen fibers; as an effective component of bone marrow, it is also involved in the proliferation, metastasis, and angiogenesis of a variety of tumor cells [23]. Type I collagen gene deficiency can promote the metastasis of breast cancer cells. Want3a/*β*-Mrtf-a silencing in the catenin pathway can reduce the binding of acetylated histone H3K9 and RNA polymerase on the COL1A1 promoter and reduce the expression of COL1A1, while the transcription factor Osterix can directly bind to the COL1A1 promoter and upregulate the expression of COL1A1 [24]. COL1A1 was reported to be involved in the differentiation and metastasis of human bladder cancer [25]. Liu et al. reported that COL1A1 can mediate the metastasis of breast cancer, which might become the potential target for breast cancer [23].

In conclusion, the present study screened 46 DEMs in GSE17681 and 1029 DEGs in GSE18842 and identified six hub genes related to lung cancer, including mTOR, NF1, CHD7, ETS1, IL-6, and COL1A1, which might be potential targets for lung cancer. This study used bioinformatics to find the key to lung cancer, provided important potential targets for early diagnosis and prognosis of lung cancer, and formulated new diagnosis and treatment strategies for patients. However, the limitation of this study lies in the lack of experimental evidence for the key genes screened. The next line of research can continue to explore the impact of these key genes on lung cancer in vivo and in vitro experiments.

## Data Availability

The data used to support the findings of this study are available upon reasonable request from the corresponding author.

## References

[1] Zhen Q., Gao L., Wang R. F. (2018). LncRNA DANC promotes lung cancer by sequestering miR-216a. *Cancer Control*.

[2] Bade B. C., Dela Cruz C. S. (2020). Lung cancer 2020: epidemiology, etiology, and prevention. *Clinics in Chest Medicine*.

[3] Wu F., Wang L., Zhou C. (2021). Lung cancer in China: current and prospect. *Current Opinion in Oncology*.

[4] Herbst R. S., Morgensztern D., Boshoff C. (2018). The biology and management of non-small cell lung cancer. *Nature*.

[5] Zhao J., Guo C., Ma Z., Liu H., Yang C., Li S. (2020). Identification of a novel gene expression signature associated with overall survival in patients with lung adenocarcinoma: a comprehensive analysis based on TCGA and GEO databases. *Lung Cancer*.

[6] Zhang R., Ma A. (2021). High expression of MYEOV reflects poor prognosis in non-small cell lung cancer. *Gene*.

[7] Zhang W., Zhang Q., Che L. (2022). Using biological information to analyze potential miRNA-mRNA regulatory networks in the plasma of patients with non-small cell lung cancer. *BMC Cancer*.

[8] Wu S. G., Shih J. Y. (2018). Management of acquired resistance to EGFR TKI-targeted therapy in advanced non-small cell lung cancer. *Molecular Cancer*.

[9] Herbst R. S., Prager D., Hermann R. (2005). TRIBUTE: a phase III trial of erlotinib hydrochloride (OSI-774) combined with carboplatin and paclitaxel chemotherapy in advanced non-small-cell lung cancer. *Journal of Clinical Oncology*.

[10] Rodriguez-Canales J., Parra-Cuentas E., Wistuba I. I. (2016). Diagnosis and molecular classification of lung cancer. *Cancer Treatment and Research*.

[11] Mao Y., Xue P., Li L. (2019). Bioinformatics analysis of mRNA and miRNA microarray to identify the key miRNA-gene pairs in small-cell lung cancer. *Molecular Medicine Reports*.

[12] Wangpaichitr M., Wu C., You M. (2008). Inhibition of mTOR restores cisplatin sensitivity through down-regulation of growth and anti-apoptotic proteins. *European Journal of Pharmacology*.

[13] Conde E., Angulo B., Tang M. (2006). Molecular context of the EGFR mutations: evidence for the activation of mTOR/S6K signaling. *Clinical Cancer Research*.

[14] Dudek H., Datta S. R., Franke T. F. (1997). Regulation of neuronal survival by the serine-threonine protein kinase Akt. *Science*.

[15] Radomska K. J., Coulpier F., Gresset A. (2019). Cellular origin, tumor progression, and pathogenic mechanisms of cutaneous neurofibromas revealed by mice with Nf1 knockout in boundary cap cells. *Cancer Discovery*.

[16] Zhen T., Kwon E. M., Zhao L. (2017). Chd7 deficiency delays leukemogenesis in mice induced by Cbfb-MYH11. *Blood*.

[17] Pleasance E. D., Stephens P. J., O’Meara S. (2010). A small-cell lung cancer genome with complex signatures of tobacco exposure. *Nature*.

[18] Colbert L. E., Petrova A. V., Fisher S. B. (2014). CHD7 expression predicts survival outcomes in patients with resected pancreatic cancer. *Cancer Research*.

[19] Nazir S. U., Kumar R., Singh A. (2019). Breast cancer invasion and progression by MMP-9 through Ets-1 transcription factor. *Gene*.

[20] Furlan A., Vercamer C., Heliot L., Wernert N., Desbiens X., Pourtier A. (2019). Ets-1 drives breast cancer cell angiogenic potential and interactions between breast cancer and endothelial cells. *International Journal of Oncology*.

[21] Okuducu A. F., Zils U., Michaelis S. A. M., Michaelides S., von Deimling A. (2006). Ets-1 is up-regulated together with its target gene products matrix metalloproteinase-2 and matrix metalloproteinase-9 in atypical and anaplastic meningiomas. *Histopathology*.

[22] Kaur S., Bansal Y., Kumar R., Bansal G. (2020). A panoramic review of IL-6: structure, pathophysiological roles and inhibitors. *Bioorganic & Medicinal Chemistry*.

[23] Liu J., Shen J. X., Wu H. T. (2018). Collagen 1A1 (COL1A1) promotes metastasis of breast cancer and is a potential therapeutic target. *Discovery Medicine*.

[24] Geng Q., Shen Z., Li L., Zhao J. (2021). COL1A1 is a prognostic biomarker and correlated with immune infiltrates in lung cancer. *PeerJ*.

[25] Mori K., Enokida H., Kagara I. (2009). CpG hypermethylation of collagen type I alpha 2 contributes to proliferation and migration activity of human bladder cancer. *International Journal of Oncology*.

